# Ensuring quality in contextualised cancer management guidelines for resource-constraint settings: using a systematic approach

**DOI:** 10.1136/bmjgh-2022-009584

**Published:** 2022-08-19

**Authors:** Manju Sengar, C S Pramesh, Abha Mehndiratta, Sudeep Shah, Anusheel Munshi, D K Vijaykumar, Ajay Puri, Beela Mathew, Ramandeep Singh Arora, Priya Kumari T, Kedar Deodhar, Santosh Menon, Sridhar Epari, Omshree Shetty, Francoise Cluzeau

**Affiliations:** 1 Tata Memorial Centre, Mumbai, India; 2 Homi Bhabha National Institute, Mumbai, India; 3 Center for Global Development, Washington, Columbia, USA; 4 P.D. Hinduja Hospital and Medical Research Centre, Mumbai, India; 5 Manipal Hospital, Delhi, India; 6 Amrita Institute of Medical Sciences, Cochin, Kerala, India; 7 Regional Cancer Centre, Thiruvananthapuram, Kerala, India; 8 Max Institute of Cancer Care, Max Super Speciality Hospital, New Delhi, New Delhi, India

**Keywords:** Cancer, Health systems

## Abstract

To address the wide variation in access to cancer care in India requires strengthening of infrastructure, trained oncology workforce, and minimisation of out-of-pocket expenditures. However, even with major investments, it is unlikely to achieve the same level of infrastructure and expertise across the country. Therefore, a resource stratified approach driven by evidence-based and contextualised clinical guidelines is the need of the hour. The National Cancer Grid has been at the forefront of delivery of standardised cancer care through several of its initiatives, including the resource-stratified guidelines. Development of new guidelines is resource and time intensive, which may not be feasible and can delay the implementation. Adaptation of the existing standard guidelines using the transparent and well-documented methodology with involvement of all stakeholders can be one of the most reasonable pathways. However, the adaptation should be done keeping in mind the context, resource availability, budget impact, investment needed for implementation and acceptability by clinicians, patients, policymakers, and other stakeholders. The present paper provides the framework for systematically developing guidelines through adaptation and contextualisation. The process can be used for other health conditions in resource-constraint settings.

**Summary box**

- High-quality, contextual and easily applicable clinical guidelines have the potential to improve the quality of care in oncology.
- Representation, participation and feedback from all stakeholders lead to transparency and better adoption of guidelines.
- Adaptation of existing guidelines using a well-documented process can help the development of context-specific guidelines with optimal use of resources.
- The resource-stratified National Cancer Grid (NCG) guidelines lend themselves for wider application in countries like India, which have a broad spectrum of expertise and infrastructure across different healthcare settings.
- The NCG guideline manual can serve as a framework for guidelines for other diseases.

## Introduction

Cancer is one of the major non-communicable diseases and a huge health burden in India. Over seven million people are estimated to die of cancer every year, accounting for 9% of all causes of death nationally and 8% of global cancer deaths.^1 2^ This high mortality has been attributed to advanced stage of disease at presentation, lack of awareness about symptoms and risk factors, stigma about cancer in the community, poor access to services and challenges with affordability.^3–5^Approximately, 50%–70% of patients present with advanced stages of cancer for their first consultation with physicians.^6^ Cancer management is often inadequate due to limited health system infrastructure, scarcity of trained oncologists and patients’ inability to afford cancer treatment.^7^ In addition, the burden of cancer and cost of cancer treatment are disproportionately high in India compared with the other diseases as a whole. Out-of-pocket payments are often prohibitively high, leading to catastrophic consequences for patients and their family.^5 8^ These factors present major hurdles for India in achieving Universal Health Care (UHC). To address these challenges, the Government of India through Department of Atomic Energy has established a network of cancer centres, the National Cancer Grid (NCG) in 2013 with the mandate of developing uniform standard of cancer care, building trained oncology workforce and developing cost-effective solutions for prevention and treatment of cancer.^9^

Another step towards UHC is the creation of the Ayushman Bharat Pradhan Mantri Jan Arogya Yojana (AB-PMJAY), the largest overnment-funded health insurance scheme in the world, which provides a cover of 5 lac INR to the vulnerable entitled families at the point of care for approved health benefit packages (HBP).^10 11^ The central focus of the National Health Authority (NHA), the administrative body governing the AB-PMJAY, is the delivery of universal quality care to its beneficiaries. The NHA is actively deploying a multipronged approach to embed quality within the scheme. Among these are the use of clinical guidelines in designing and implementing the HBPs. A major area of cooperation between the NHA and the NCG has been in linking reimbursements for oncology packages under the AB-PMJAY scheme to resource stratified guidelines developed by the NCG.^12^ Given the potential impact of NCG guidelines in improving the quality of care across the country through the AB-PMJAY, it is highly relevant that these guidelines should be evidence-based, feasible on the ground, transparent in the process of development with inputs from all the stakeholders.

This paper describes a framework for developing and contextualising clinical guidelines for cancer in India under the NCG that can potentially be applied to other diseases and other resource-conscious settings. We examine how international principles and adaptive approaches can be used within this framework, reflecting on the current state of guideline development and adaptation. Throughout the paper, we discuss how this adaptation framework links with the healthcare quality improvement efforts in the AB-PMJAY in India.

## Standardising and improving quality of cancer care through the NCG

The NCG is a network of over 260 major cancer centres, research institutes, patient groups, and charitable institutions across India, funded by the Government of India through the Department of Atomic Energy and its grant-in-aid institution, the Tata Memorial Centre (https://tmc.gov.in/ncg/). The members of the NCG are responsible for over 60% of cancer care delivery in India. One of the NCG objectives is to establish uniform standards for the prevention, diagnosis and treatment of cancer, by adopting implementable evidence-based management guidelines that are developed by different groups within the network (https://tmc.gov.in/ncg/index.php/guidelines/draft-guidelines-2020). In doing so, the NCG attempts to reduce disparities in the standards of patient care in various geographic regions of India, identifying cost-effective management strategies which can be implemented in all centres and can be accessed by all.

NCG guidelines are relevant for both public and private cancer providers: clinicians, managers, payers (health insurers) and also to patients. Linking to the AB-PMJAY scheme, the guidelines aim to strengthen the delivery of cancer services under the scheme by providing guidance to standardise and optimise beneficiaries’ care and to support better decision-making during disease management and reimbursements. Recently, the NCG signed a memorandum of understanding with the NHA to develop guidelines on patient care for cancer under the AB-PMJAY.^13^

## A framework for developing and contextualising guidelines for India

Differences in cancer epidemiology, disparities in resources, expertise, access, and affordability preclude the possibility of adopting the international guidelines to India and call for either developing or adapting and contextualising the existing standard guidelines. Clinical guidelines can only bring benefits if they have been rigorously developed, evidence-based, contextually sound and acceptable by clinicians who are aware of their existence for incorporation into clinical practice.^14 15^ To ensure that the NCG guidelines are of high quality and are developed in a consistent manner across its groups, the NCG has produced a manual that describes the methods and processes used during the development phase (https://tmc.gov.in/ncg/index.php/guidelines/guidelines-manual1). The manual also includes practical tools for ready use by developers. To increase buy-in from its members, the NCG prepared the manual through two rounds of consultation with its guideline development group (GDG) coordinators. Their views were considered and incorporated into the final version based on the supporting evidence.

**Figure 1:**
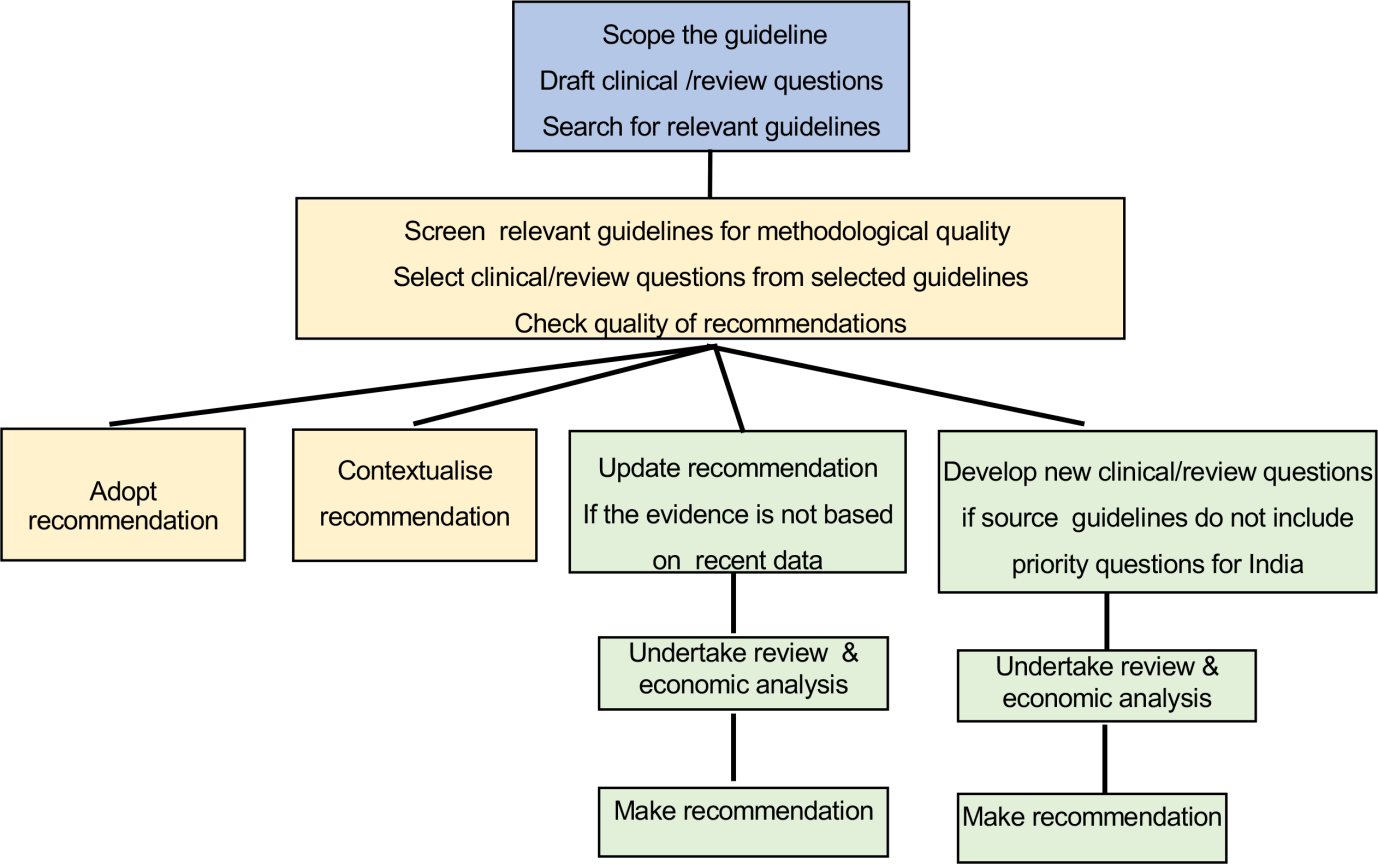
Process for developing and contextualising NCG guidelines for India. NCG, national cancer grid.

Developing de novo clinical guidelines is costly, time-consuming and requires dedicated teams of methodologists or experts,^16^ which may pose a challenge for NCG. From the outset, the NCG opted to use existing high-quality guidelines and contextualise them to the Indian context rather than developing de novo guidelines. This approach is increasingly recommended and used in low and middle-income countries with limited resources.^17 18^ Different approaches have been reported on how to adapt clinical guidelines and contextualising existing high-quality guidelines to make recommendations relevant to local contexts.^19–22^ The ‘RIGHT Adapt’ framework developed by an international collaboration has undergone the most systematic and rigorous development.^23^ The NCG used this framework, through a transparent and inclusive process, to ensure the timely development of workable guidelines in India at this nascent stage of the process and ‘that the final recommendations address specific health questions relevant to the context of use, and address the needs, priorities, legislation, policies and resources in the target setting without undermining the validity of the target recommendations’.^17^ The overarching process of using and adapting existing guidelines is shown in figure 1.

### Engaging stakeholders in the development process

The importance of including all stakeholders in the development of clinical guidelines is widely recognised^24 25^ and is acknowledged in the NCG guidelines. Stakeholders include all those who have a legitimate interest in a guideline and include not only healthcare professionals, patients and caregivers but also the insurers, managers, policymakers and manufacturers who can send their suggestions after the draft guidelines are made available on the website for comments. Their engagement is justified for several reasons, including limitations of evidence, principles of transparency and democracy, ownership and potential policy implications. Therefore, guidelines need to consider explicitly the values and preferences of all these relevant stakeholders and to provide opportunities to engage in processes that consider and integrate their values into the development of guideline recommendations.

Stakeholders have a role to play at different points of guideline development, but their involvement can be complex. In India, this is amplified by the highly diverse healthcare system and the wide range of stakeholders that can potentially be involved. In recognition, the NCG guideline manual contains guidance on the contribution stakeholders can make, whether this is as members of the GDG and making decisions or in providing comments through consultation on draft recommendations. The role of the GDG Chair is highlighted to ensure that the group works collaboratively,^26^ and all members contribute in reaching final recommendations, whether through informal consensus or using formal voting.^27^ Declaration of conflict of interests is the key in this process.^28^

Given the huge task of developing a guideline, it is particularly important for the GDG to identify from the outset the areas of focus.^29^

### Developing scope and contextualising existing guidelines for India

The NCG manual advocates a scoping phase where key priorities are defined with highest potential impact. The scoping document includes the draft clinical/review questions and provides a clear and transparent structure to guide the GDG.^30^ These questions consider efficacy, safety, quality of life, economic information and health services utilisation. The criteria for key review questions to be included in the scope are highlighted in box 1.

Box 1 **Criteria for selection of key review questions**

- Likely to have high impact on key outcomes.
- Likely to have high impact on reducing variation in cancer care and outcome in India.
- Relate to an intervention that is not part of routine cancer care in India.
- Require major changes in service delivery.
- Are cost-effective.
- Expected to be implemented under the Health Benefits Package of the Ayushman Bharat Pradhan Mantri Jan Arogya Yojana (AB-PMJAY) Scheme.

It informs the search for existing guidelines that are relevant for adaptation and stimulates the initial dialogue among the stakeholders.

Once potential relevant existing guidelines (source guidelines) have been identified relevant to the scope, they are screened for their methodological quality.^31^ These source guidelines have a full document that contains, as a minimum, the clinical/review questions, details of search strategies, evidence reviews and their summaries, including Grading of Recommendations, Assessment, Development and Evaluations (GRADE) tables that establish the quality of the evidence and the degree of confidence in the estimates of effect.^32^ Equally important is the link between evidence and recommendations. The higher the quality of the evidence from the source recommendation, the more confidence the GDG has in making a recommendation. This ensures that each recommendation links with the evidence and there is a clear rationale for how the final decisions were reached.^33^

However, high quality of evidence alone is not sufficient for adoption. It also needs to be applicable and acceptable in practice, relating to the cultural and organisational context, the availability and organisation of health services, expertise and resources, as well as population characteristics, beliefs and value judgments. These variables are particularly important when adapting guidelines for culturally sensitive interventions and technological innovations.^34^ The applicability of guideline recommendations is closely linked to economic factors and available resources.^35^ Economic evaluation can be used to ensure that the most cost-effective interventions are selected focusing health services on high-priority interventions based on the best available evidence.^36^ Reduction in variation in cancer care ensures consistent and equitable treatment and also offers the opportunity to achieve economies of scale in the procurement and pricing of commodities, thus reaching a larger patient base. However, even if an intervention is cost-effective, it is still necessary to determine whether it is affordable so that introducing clinical guideline recommendations into practice does not adversely affect the long-term financial stability of the health system.^37^ This is particularly relevant in defining HBPs. Budget impact analysis (BIA) that estimates the financial consequences of the adoption and implementation of a new healthcare intervention in a healthcare setting is needed. It captures the financial benefit of interventions that are costly to implement but save money over the long term.^38^

From a policymakers’ perspective, both economic analysis and BIA inform the treatments that are made routinely accessible in India under the AB-PMJAY scheme, where there is a limit of INR 500 000 per family per year. Oncology care is usually expensive, and, therefore, difficult decisions on trade-offs between treatment options need to be made to ensure that the care remains within budget and provides the greatest health impact without compromising the acceptable standard of care.^39^

## Making decisions

Making recommendations is a challenging part of the NCG guideline development process because it involves the GDG reaching decisions and coming to its final conclusions, considering a range of evidence from multiple sources and other factors that are specific to the Indian health context. In addition to the quality of evidence and financial and economic considerations already noted, the WHO-INTEGRATE framework identifies other dimensions relevant to decision making and formulation of recommendations. Among others, these include balance of health benefits and harms, health equity, equality and non-discrimination, feasibility and health system considerations.^40^ Box 2 provides some of the examples to highlight the factors taken in consideration while adapting the international guidelines.

Box 2 **Examples of making recommendation in National Cancer Grid (NCG) guidelines for non-small cell lung cancer (NSCLC) and HER-2-positive breast cancer adjuvant therapy**

**NSCLC staging**

NICE guidelines: Ensure that all people with lung cancer who could potentially have treatment with curative intent are offered positron-emission tomography CT (PET-CT) before treatment.

NCG guidelines: As many centres who treat non-small cell lung cancer across the country may not have PET-CT available to them, the use of CECT chest, abdomen and pelvis was recommended as the essential modality for staging, while PET-CT was considered ‘optimal’.

**Her-2-positive breast cancer adjuvant therapy**

NICE guidelines: Offer adjuvant trastuzumab for people with T1c and above HER2-positive invasive breast cancer, given at 3-week intervals for 1 year in combination with surgery, chemotherapy and radiotherapy as appropriate.

NCG guidelines: In view of high out of pocket expenditure resulting poor access to trastuzumab, 1-year course would not feasible. Based on the cost-effectiveness analysis from India and the meta-analysis confirming non-inferiority of short-course trastuzumab to 1 year of therapy.^49^ NCG guideliness recommend short-course trastuzumab for adjuvant therapy in Her-2-positive breast cancer.

### Balancing benefits and harms

Moving from evidence to recommendations involves weighing up the magnitude and importance of the benefits and harms of an intervention and also the potential for unintended consequences.^41^ To make these judgments, the GDG needs to appreciate how substantial the expected benefits and adverse effects of the intervention are likely to be in practice. Equally, the economic impact of an intervention needs to be evaluated with respect to the entire population, other health conditions, and the health budget. Detailed tables of the clinical and economic evidence are essential tools to help the GDG make such decisions.

### Considering health equity

In making decisions, the NCG GDGs acknowledge the potential impact their recommendations could have on health inequality due to differences in socioeconomic status and geographical region. All efforts will be made based on the available methods^42^ to put care pathways in place to prevent such inequalities within the country.

### Feasibility of implementation

Assessing the feasibility of implementing the recommendations into practice is of paramount importance. Having already addressed these issues during the planning and scoping stage, GDGs need to consider the extent of change in practice that will be needed to implement a recommendation in cancer centres: staffing, equipment, service organisation; at the national level, policy levers, information and service infrastructure, supplies and funding streams and the possible need for a carefully controlled implementation with, for example, training programmes, gradual reorganisation of services and potential capital investment.^43^ These factors are especially relevant in deciding whether the recommendations should inform the HBP.

## The final stage of guideline adaptation

The factors discussed above are especially relevant when the GDG decides on the recommendations from the existing guidelines they are using in the adaptation process and to ensure that they are appropriate for cancer care in India. In their deliberations, the GDG weighs up three options: *Adopt* a recommendation, *contextualise* a recommendation to the Indian context and *Update a recommendation*. These three options are described in table 1.

**Table 1:** Process of adaptation and contextualising the recommendations from the internationally accepted guidelines

Recommendation option	Definition
Adopt a recommendation	Reproducing a recommendation verbatim from the source guideline. Recommendations are adopted when they can be applied directly, without any changes, to the Indian context
Contextualise a recommendation to India	Reproducing a recommendation verbatim from the source guideline but adding a commentary about local context conditions needed for implementing the recommendation. Contextual points can include comments relating locally appropriate alternative methods of intervention delivery, system issues that would need to be considered to be implemented in the current India care system
Update a recommendation	Arises when the evidence underpinning a recommendation needs updating in the light of recent research. This will require updating the literature search and evidence assessment if needed, and may lead to a new recommendation

When a source guideline does not cover an area of priority identified during the scoping of the NCG guideline, the GDG may decide to develop a new clinical/review question. In this case, this requires a new review of evidence according to the ‘de novo guideline development process’.

## Resource-stratified recommendations

It is generally accepted that some guideline recommendations are stronger than others, for example, based on the certainty of benefit from the evidence and whether others (guideline users) would reach similar conclusions and that this should be conveyed to users.^19^ However, there is no universally accepted approach about how the strength of recommendations should be represented or classified. In the NCG guidelines, the strength of recommendations is represented as ‘resource-stratified recommendations’ that consider the clinical evidence, equity, costs, and also implementation considerations. The NCG categorises the guideline recommendations into three groups: ‘essential’, ‘optimal’ and ‘optional’. These are described in table 2. This classification is specifically designed for implementing NCG guidelines in India and links to the AB-PMJAY HBP. The classification has important implications for guiding practice that will inform preauthorisation and claims processing where documentation will be required. The GDG member selection process ensured representation from all the tiers with diverse resources and infrastructure. This coupled with multiple rounds of consultation within the GDG based on choosing wisely framework helped^44^ to stratify the interventions and select the minimum standard of care (essential category).

**Table 2:** Levels of resource stratification in NCG guidelines

Classification	Definition
Essential	Recommendations based on the evidence, practicality (wide availability of expertise and infrastructure) as well as the cost of treatment and the value it offers. If centres do not have the capabilities to implement these, they should refer patients to a higher centre
Optimal	Recommendations based on both evidence as well as cost-effectiveness, but may not be widely available because of issues with expertise and infrastructure
Optional	Recommendations that reflect the state of the art, and are based purely on the available evidence with no consideration for cost-effectiveness

NCG, national cancer grid.

## Conclusion

The NCG is a network of cancer centres that collectively provides care to a majority of the patients with cancer in India and covers millions of patients. Improving the quality, access, and affordability of cancer care services to patients is at the core of the NCG mission, whether the care is delivered in the private or public healthcare setting. One of its main initiatives to achieve this is by developing clinical guidelines that are evidence-informed, inclusive, context-stratified, and realistic for India and that can be developed within the technical and financial resources of its programme. The NCG’s pragmatic guideline adaptation framework attempts to produce guidelines that follow the international principles of robust clinical guideline development, while avoiding the lengthy and costly process of de-novo guideline production of well-resourced national guideline programmes.^45^ The size and complexity of the Indian healthcare system are unique and the process of making guideline recommendations for India can only be made realistically within India, balancing all the factors that are distinctive to the country. Not all clinical reviews and questions from source guidelines will be applicable to the Indian context. Conversely, there are priorities for India that have not been addressed in existing guidelines and will require new reviews and health technology assessment (HTA). India is building a strong basis for HTA through the HTAIn programme and academic centres of excellence are developing health economics capacity across the country,^46^ using innovative approaches such as adaptive HTA.^47^

An important aspect of the NCG Guidelines programme is its link with the AB-PMJAY Scheme and the significant contribution it can make to its quality agenda. In the context of redesigning the healthcare system, NCG guidelines help standardise cancer care through implementing the health benefits packages and the use of strategic purchasing contracting and provider payments, helping India to achieve UHC.^48^

The guideline adaptation framework of the NCG is directly applicable to other disease areas. The principles discussed in this paper, and that are embedded in the approach are universally recognised and are relevant across the whole spectrum of diseases and care.^23^

The NCG guideline development journey is still new and is likely to encounter challenges as it evolves. The buy-in from clinicians across India is crucial to getting the guidelines implemented in practice, as are other key stakeholders, including policymakers and patients. The concept of evidence-informed practice and how guidelines can inform decisions are still unfamiliar to many in India. However, a process of guideline development that reaches out to users includes them in the planning and decision-making and is transparent can bolster knowledge and acceptance on a wider scale.

Clinical guidelines are increasingly playing a key role in improving the quality of care and access to services for millions of people across India. The NCG is spearheading the development of clinical cancer guidelines that can be used at the point of delivery across its centres. Importantly, they can serve to inform the HBP and reimbursement of interventions under the AB-PMJAY scheme, benefiting millions of patients and their families. The NCG guideline adaptation framework could easily be used across other major diseases across India and contribute to its journey to UHC.

## Data Availability

Data sharing not applicable as no datasets generated and/or analysed for this study.
